# Distance Neurological Supervision Using Telestroke Does Not Increase Door-to-Needle Time in Acute Ischemic Stroke Management: The Experience of Two Regional Stroke Units

**DOI:** 10.3389/fneur.2021.616620

**Published:** 2021-03-19

**Authors:** Radhika Sood, Jean-Marie Annoni, Andrea M. Humm, Ettore Accolla, Olivier Bill, Guillermo Toledo Sotomayor, Julien Niederhauser, Friedrich Medlin

**Affiliations:** ^1^Neurology Unit, Hôpital Fribourgeois, Fribourg, Switzerland; ^2^Department of Neurosciences, Fribourg University, Fribourg, Switzerland; ^3^Stroke Unit, Groupement Hospitalier de l'Ouest Lémanique, Nyon, Switzerland

**Keywords:** thrombolysis, neurologist, door-to-needle time, acute ischemic stroke, teleradiology for acute stroke management

## Abstract

**Background and Aims:** Timely administration of recombinant tissue plasminogen activator (r-tPA) improves clinical outcomes in acute ischemic stroke patients. This study aims to explore the influence of the systematic presence on site of a neurologist compared with telestroke management on door-to-needle time in acute ischemic stroke outside of working hours (OWH).

**Methods:** This retrospective cohort study included all r-tPA-treated patients in the emergency rooms of two Swiss stroke units, Nyon Hospital [Groupement Hospitalier de l'Ouest Lémanique (GHOL)] and Fribourg Hospital [Hôpital de Fribourg (HFR)], between February 2014 and September 2018. Door-to-needle time was analyzed for patients admitted during working hours (WH' weekdays 08:00–18:00) and OWH (weekdays 18:00–08:00, weekends, and public holidays). The latter was compared between centers; OWH, every patient was evaluated prior to thrombolysis by a neurologist on site in GHOL, while HFR adopted distance neurological supervision with teleradiology, performed by telephone evaluation of relevant clinical information with online real-time access to brain imaging.

**Results:** Data were analyzed for 157 patients in HFR and 101 patients in GHOL. No statistically significant differences in baseline characteristics were found for the 258 r-tPA-treated acute ischemic stroke patients, in terms of age, gender, cardiovascular risk factors (hypertension, diabetes, and atrial fibrillation), and pre-Modified Rankin Scale (pre-mRs) between centers, with the exception of smoking and anticoagulation status. Patients in HFR presented with more severe strokes {median National Institutes of Health Stroke Scale (NIHSS) [6 (SD 6.88) (GHOL), 8 (SD 6.98) (HFR), *p* = 0.005]}. No significant differences in baseline characteristics were found as per admission time independently of the center. Door-to-needle time was significantly longer in the HFR cohort when compared with GHOL, irrespective of admission time. Both centers demonstrated significantly longer median door-to-needle time OWH. However, analysis of the door-to-needle time differences between WH and OWH showed no significant interaction using robust ANCOVA WRS2 analysis (*p* = 0.952) and a Bayesian model (BF01 = 3.97).

**Conclusions:** On-site systematic evaluation by a neurologist did not appear to influence door-to-needle time OWH, suggesting distance supervision may be time-efficient in thrombolysis. This supports existing prospective studies in hyperacute telestroke management. The relevance lies in optimizing resource use considering the increasing demand for emergency neurological management.

## Introduction

Improved clinical outcomes in acute ischemic stroke (AIS) are associated with timely administration of intravenous recombinant tissue plasminogen activator (r-tPA) (1, 2). Shorter door-to-needle times (DNTs) reduce ischemic insult to brain tissue through repermeabilization of blood vessels. Fonarow et al. demonstrated a 5% reduction in in-hospital mortality with every 15-min reduction in DNT (3).

Following a paradigm shift in 2015, endovascular treatment (EVT) is recognized as the standard of care for selected AIS patients with proximal occlusion of the anterior circulation (4). The prompt recognition of proximal vessel occlusion, rapid initiation of intravenous thrombolysis (IVT), and further referral for mechanical revascularization is of prime importance. In Switzerland, the management of ~50% of AIS patients is shared between 10 stroke centers and 13 stroke units (5).

With the aging of the global population and the growing indication for AIS revascularization, demand for emergency stroke management is increasing (6). Meanwhile, the supply of neurologists, although ensuing an upward trend, is not following at the same allure (7). The importance of the implication of a neurologist is recognized in stroke management. While specialized neurological evaluation of AIS patients has been shown to reduce the length of hospital stay (8), it is also important to consider the role of the neurologist in hyperacute stroke management. Regarding acute stroke management outside of working hours (OWH), local stroke units and centers in Switzerland adopt different approaches to neurological presence (physical or through distance supervision). Comparison of different systems is rendered possible by the systematic training of emergency department staff and homogenous protocolled pre-hospital stroke care found in the Swiss stroke units considered.

The purpose of this study is to explore the influence of the systematic presence on site of neurologists compared with telestroke management, defined in this study as using telephone and teleradiology without video clinical evaluation, on DNT in AIS patients.

## Methods

This study is a two-center retrospective cohort analysis performed on AIS patients treated with r-tPA in the emergency rooms of two Swiss stroke units. Fribourg Hospital [Hôpital de Fribourg (HFR)] and Nyon Hospital [Groupement Hospitalier de l'Ouest Lémanique (GHOL)] receive 540 patients annually in their corresponding emergency rooms (~320 and 220 patients, respectively). Data were extracted from local stroke registries and analyzed for all patients treated consecutively with r-tPA between February 2014 and September 2018. Local stroke registries were kept by doctors involved in patient management, who recorded information soon after the event. All patients who received thrombolytic therapy in this timeframe were included. Decisions pertaining to the administration of IVT were based on the European Stroke Organization guidelines, updated following the Karolinska Stroke Update meeting in 2008 (9).

AIS management protocol changed in GHOL in 2019, when this stroke unit began implementing distance supervision OWH. Observation of other systems functioning in a relatively close vicinity, such as that of HFR, appeared to show systems operating adequately through use of distance supervision. The decision to implement distance supervision was based on the latter, along with the difficulty in terms of human resources in ensuring 24-h on-site neurological presence. Duties in GHOL were shared between three Swiss certified neurological specialists.

In both centers, initial patient evaluation was performed by emergency room physicians. The team of health-care professionals involved included an emergency room nurse, a junior doctor, and a specialist registrar. All doctors in both centers received at the minimum biannual National Institutes of Health Stroke Scale (NIHSS)-oriented stroke management training provided by a stroke specialist.

While stroke care systems differ between the two stroke units, a common factor is that emergency room duties are shared between few neurologists. All of the involved neurologists were Swiss board certified and experienced in stroke management. Neurological involvement was similar in both centers during WH (working hours) but differed OWH. Every AIS patient eligible for IVT was evaluated prior to r-tPA administration by a neurologist in GHOL independent of admission time, while the systematic presence of a neurologist was not obligatory OWH in HFR. HFR offered distance supervision with teleradiology OWH, based on telephone communication of relevant patient information, respecting a detailed stroke protocol and online real-time access to brain imaging. The latter was obtained through computed tomography (CT) imaging, located in the Emergency Department in GHOL and in the Radiology Department in HFR.

DNT is evaluated as the primary outcome and compared as per the admission time (WH or OWH). WH were defined as 08:00 to 18:00 on weekdays and OWH as 18:00 to 08:00, weekends, and public holidays. It is significant to note that pre-hospital patient management in Switzerland involves standard ambulances and that mobile stroke units do not exist in the areas considered. In GHOL, upon ambulance intervention, the neurologist was alerted of the suspected stroke patient and required to arrive on site irrespective of admission time. In HFR, the neurologist was alerted by a stroke code, initiated at the time of patient arrival, following initial emergency room doctor triage. During WH, the neurologist immediately evaluated the patient together with the emergency room physicians. OWH, the emergency physician presented the relevant clinical information prior to imaging by telephone to the neurologist who followed the imaging by means of teleradiology and accordingly made a therapeutic decision. The latter did not involve any visual clinical patient evaluation using cameras. DNT is defined as the time (in minutes) from arrival in the hospital to r-tPA bolus administration. In the exceptional case of in-hospital strokes, the latter is defined as the time of symptom onset to r-tPA bolus administration.

Amongst admitted AIS patients during the study period, HFR recorded annual IVT rates of 16%, compared with 36% in GHOL. Furthermore, 6% of patients were referred from each site for EVT, annually. GHOL referred patients to Lausanne University Hospital, while HFR referred patients to either Lausanne University Hospital or Bern University Hospital depending on the patient's origin; the latter subgroup was predominant in the HFR population.

## Statistical Analysis

Dichotomous data are represented as percentages and continuous data as means or medians with standard deviations of means. Interquartile ranges are presented for data that is non-normally distributed. Values are represented as percentages, unless otherwise indicated. The *p*-value is determined by use of the independent samples *T*-test for age, NIHSS on admission, and pre-Modified Rankin Scale (pre-mRs). The χ^2^-test is used for categorical variables. Extent in Rankin shift (difference between mRs on admission and mRs 3 months following AIS) is represented as an ordinal variable. The range of mRs scores is represented as Grotta bars (a paired horizontal stacked bar graph).

Statistical significance was set at *p* < 0.05. All statistical analyses were carried out using https://www.jamovi.org/statistics. The study was developed in conjunction with the STROBE guidelines.

## Results

### Baseline Characteristics

As shown in Table 1, a total of 258 AIS patients were included between February 2014 and September 2018, of whom 54.7% were male and 50.8% were treated during WH. The mean age was 72.3 years; the median NIHSS on admission was seven and 56 patients (21.7%) were sent to the adjacent comprehensive Stroke Center for potential EVT. Analyses revealed differences in baseline demographics between centers (Table 1). Smoking was predominant in the HFR cohort (*p* = 0.047). Differences in pre-hospital triage between the two centers explain the significant difference in anticoagulation status; initial pre-hospital algorithms in the canton of Fribourg involved the immediate transfer of all anticoagulated patients with a significant neurological deficit (G-FAST score > 2) (10) in the therapeutic window of up to 24 h from symptom onset to adjacent Stroke Centers (Lausanne University Hospital or Bern University Hospital) prior to evaluation in HFR. Clinical evaluation of patients further revealed a higher NIHSS in the HFR cohort (*p* = 0.005). Of these patients, 26.1% were transferred to the adjacent stroke center for potential EVT in HFR compared with 14.9% in GHOL (*p* = 0.032), as shown in Table 1. There does not appear to be a significant difference in the proportion of patients presenting with stroke mimics treated with IVT between both centers (7% in GHOL and 5.7% in HFR, *p* = 0.697). Baseline characteristics are similar between WH and OWH; it is significant to note that no difference in stroke severity was found as per admission time, when comparing NIHSS (*p* = 0.550).

**Table 1:** Site-specific demographics and clinical characteristics; demographics and clinical characteristics as per admission time.

**Variable**	**Center**		***p*^a^**	**Admission time**		***p*^a^**	**Total (*n* = 258)**
	**GHOL (*n* = 101)**	**HFR (*n* = 157)**		**WH (*n* = 130)**	**OWH (*n* = 128)**		
Age, mean (SD) (IQR), years	73.1 (14.1) (22)	71.9 (14.8) (18)	0.523	73.9 (15) (19)	70.7 (13.9) (21)	0.076	72.3 (14.5) (19.7)
Male sex (%)	54.5	54.8	0.960	50.8	58.6	0.207	54.7
Arterial hypertensio*n* (%)	66.3	66.2	0.519	68.5	64.0	0.305	66.5
Diabetes (%)	15.8	16.6	0.263	18.5	14.0	0.394	16.3
Smoker (%)	13.9	17.2	**0.047**	14.6	17.2	0.274	15.9
Atrial fibrillation (%)	24.8	24.1	0.194	25.4	23.4	0.377	24.4
Anticoagulation (%)	6.9	0.6	**0.004**	3.07	3.13	0.982	3.1
Pre-mRs, median (SD)	0 (1.14)	0 (1.05)	0.662	0 (1.15)	0 (1.02)	0.202	0 (1.09)
NIHSS, median (SD), (IQR)	6 (6.88) (5)	8 (6.98) (11)	**0.005**	6.50 (6.88) (9)	8 (7.20) (10)	0.550	7 (7.04) (9.00)
Transfer to adjacent stroke center for potential endovascular treatment following thrombolysis (%)	14.9	26.1	**0.032**	20.0	23.4	0.503	21.7

*GHOL, Groupement Hospitalier de l'Ouest Lémanique; HFR, Hôpital de Fribourg; IQR, interquartile range; mRs, Modified Rankin Scale; NIHSS, National Institutes of Health Stroke Scale*.

a *p: Determined by use of the independent samples T-test for age, NIHSS on admission, and pre-mRs. The χ^2^-test is used for categorical variables*.

*Statistically Significant values are indicated in bold (p ≤ 0.05)*.

Table 2 shows site-specific demographics and clinical characteristics as per admission time. Thrombolysis was administered to 50 patients during working hours in GHOL (49.5%), compared with 80 patients in HFR (51%). No significant differences between the patient cohorts in each center were found irrespective of admission time.

**Table 2:** Inter-site-specific demographics and clinical characteristics according to admission time.

**Variable**	**GHOL (*****n*** = **101)**		***p*^a^**	**HFR (*****n*** = **157)**		***p*^a^**
	**WH (*n* = 50)**	**OWH (*n* = 51)**		**WH (*n* = 80)**	**OWH (*n* = 77)**	
Age, mean (SD) (IQR), years	74.1 (13.6) (52)	72.1 (14.6) (64)	0.483	73.8 (15.9) (74)	69.8 (13.4) (69)	0.088
Male sex (%)	54	55	0.557	48.8	61	0.123
Arterial hypertension (%)	62	70.6	0.364	72.5	59.7	0.076
Diabetes (%)	14	17.6	0.618	21.3	11.7	0.323
Smoker (%)	14	13.7	0.968	15	19.5	0.119
Atrial fibrillation (%)	26	23.5	0.775	25	23.4	0.525
Anticoagulatio*n* (%)	6	9.8	0.717	1.25	0	0.327
Pre-mRs, median (SD)	0 (1.22)	0 (1.05)	0.267	0 (1.11)	0 (0.997)	0.468
NIHSS, median (SD), (IQR)	7 (6.66) (26)	5 (7.13) (36)	0.558	6 (7.02) (29)	9 (6.92) (27)	0.199
Transfer to adjacent stroke center for potential endovascular treatment following thrombolysis (%)	16	13.7	0.749	22.5	29.9	0.295

*GHOL, Groupement Hospitalier de l'Ouest Lémanique; HFR, Hôpital de Fribourg; IQR, interquartile range; mRs, Modified Rankin Scale; NIHSS, National Institutes of Health Stroke Scale*.

a *p: Determined by use of the independent samples T-test for age, NIHSS on admission, and pre-mRs. The χ^2^-test is used for categorical variables*.

### Analysis of Door-to-Needle Time According to Admission Time and Center

Analyses of DNT according to admission time show a significant difference with a longer median DNT OWH compared with WH in both centers, as shown in Table 3. In GHOL, median DNT during WH was 22.0 ± 4.85 min [95% confidence interval (CI) 17.1, 26.9] vs. 30.0 ± 7.82 min [95% CI 22.2, 37.8] OWH, *p* = 0.0024. In HFR, median DNT was 42.0 ± 4.29 min [95% CI 37.7, 46.3] during WH vs. 52.0 ± 4.53 min [47.5, 56.5] OWH, *p* = 0.033. DNT is significantly longer in the HFR cohort when compared with the GHOL population.

**Table 3:** Door-to-needle time according to center (GHOL and HFR) and admission time (working hours vs. outside of working hours).

	**DNT as per admission time, median** ± **(95% CI) (SD), min**		***p*-value between WH and OWH^a^**	**Admission time × Center ANCOVA WRS2 interaction**
	**WH**	**OWH**		
GHOL (*n* = 101)	22.0 ± 4.85 (17.1, 26.9) (17.5)	30.0 ± 7.82 (22.2, 37.8) (28.5)	**0.024** (E^2^ – 0.455)	**0.952**
HFR (*n* = 157)	42.0 ± 4.29 (37.7, 46.3) (19.6)	52.0 ± 4.53 (47.5, 56.5) (20.3)	**0.033** (E^2^ – 0.344)	

*GHOL, Groupement Hospitalier de l'Ouest Lémanique; HFR, Hôpital de Fribourg; IQR, interquartile range; mRs, Modified Rankin Scale; NIHSS, National Institutes of Health Stroke Scale*.

a *p: Determined by use of the independent samples T-test, with representation of E^2^, effect size*.

*Statistically Significant values are indicated in bold (p ≤ 0.05)*.

### Analysis of Interaction of Differences in Door-to-Needle-Time According to Admission Time

Robust ANCOVA WRS2 analysis found no interaction in DNT between the center and admission time (*p* = 0.952) (11). This was further confirmed using a Bayesian model, showing a four times higher likelihood of the null hypothesis (BF01 = 3.97). This also applied when analysis was repeated following in-hospital stroke exclusion (relevant to three cases in GHOL and six cases in HFR).

**Figure 1:**
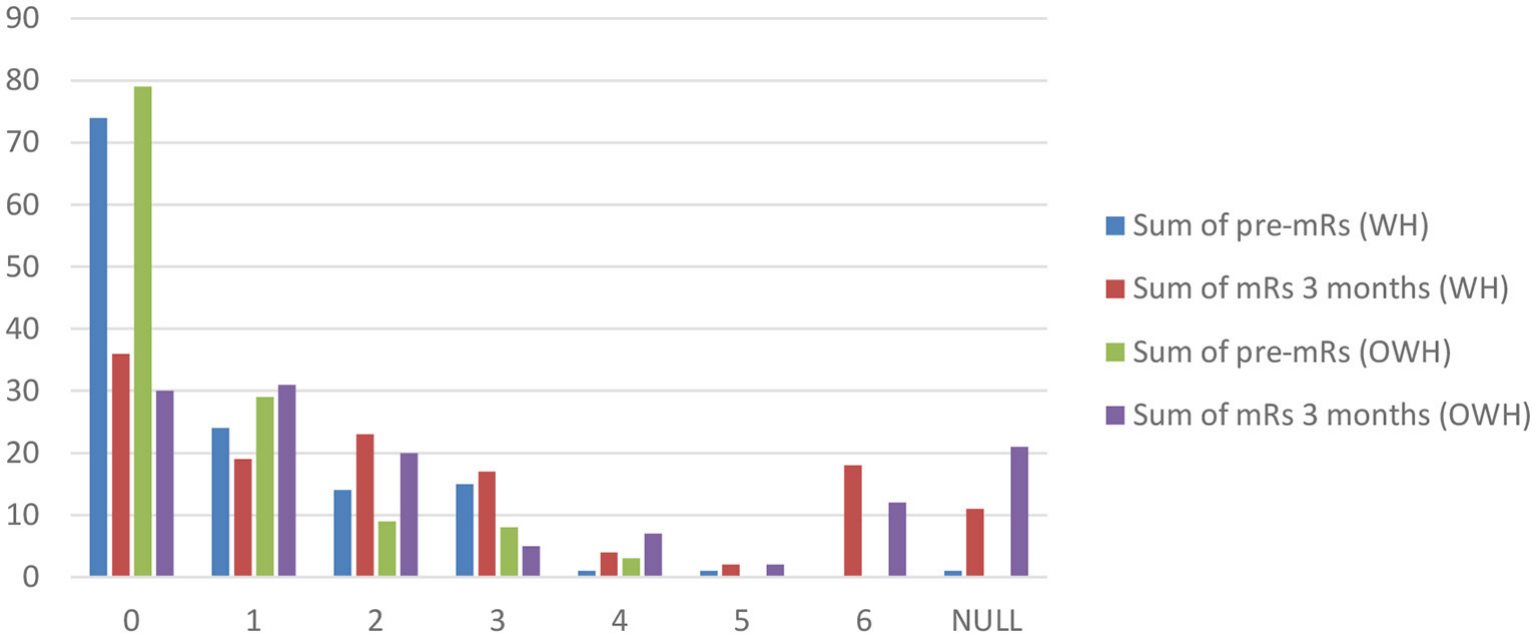
Pre-mRs compared with post-mRs evaluated 90 days following acute ischemic stroke according to admission time. mRs, Modified Rankin Scale.

The mRs is comparable independent of admission time (Table 1, *p* = 0.202). Follow-up outcomes, such as the extent in Rankin shift, that is, the difference between the 3-month mRs and mRs upon admission for AIS, are comparable in both centers as shown in Figure 1. Trends appear similar irrespective of admission time for cumulative data between both centers, except for an mRs of 1 (no significant disability) and 3 (moderate disability). Statistical significance was not analyzed.

## Discussion

Concerning the clinical question explored, this study found that while DNT is longer OWH in both centers, analysis of the interaction of differences in DNT between WH and OWH showed no significant interaction. We hypothesize that adequate communication between the remote neurologist (using teleradiology with real-time online imaging) and on-site emergency team department with the radiologist can allow for adequate administration of IVT in certified stroke units. Protocolled stroke management involving the relevant medical health-care professionals may compensate for the physical absence of the neurologist. This is highly relevant, as indicated by a survey conducted in 24 hospitals in Michigan (United States), where 65% of emergency department doctors reported feeling uncomfortable with the administration of r-tPA without prior neurological opinion (12). Distance supervision does not appear to increase DNT or increase the difference observed between pre-mRs and mRs post-ischemic event and therefore appears to be time-efficient in the management of hyperacute stroke. The use of the standardized ordinal scale, NIHSS, allows for remote evaluation through distance supervision (13)?

The administration of thrombolysis is subject to many variables, patient- and system-related, which contribute to delay. Pre-hospital delays are not addressed in this study but must be incorporated when considering clinical outcomes following stroke. This two-center analysis highlights major inter-center differences. In a study published in 2014, inter-hospital variability was shown to be responsible for 12.7% of variation in DNT (14). Logistical reasons are likely to explain the inter-hospital variation in DNT between GHOL and HFR. GHOL receives stroke patients directly in the radiology CT unit for clinical evaluation followed by immediate neuroimaging, allowing patients to bypass the emergency room, while in HFR, patients are subject to emergency room evaluation before being transported to the radiology unit. Furthermore, differences in DNT may also result from differences in patient numbers. Fewer patients were thrombolyzed in GHOL when compared with HFR for the same period.

Analysis of patient data highlights a significant difference in NIHSS between both centers: the NIHSS upon admission is lower in GHOL than HFR (*p* = 0.005, Table 1). This is interesting when considering existing literature; through retrospective analysis of patient data in a single center, Harvey et al. demonstrated that patients with mild ischemic stroke, defined as a NIHSS <5, were subject to a 10-min delay in receiving intravenous r-tPA (*p* = 0.007). Approximately 15% of the studied population had poor functional outcome at discharge (15). This tendency was not found in the results; nonetheless, intra-center NIHSS variability was not considered.

The increase in DNT OWH is in line with existing studies in the domain. Large cohort retrospective studies have shown diurnal variation in DNT, with a recognized “OWH effect” contributing to increased DNT (16–19). The trend in GHOL supports the latter to be considered, as stroke protocol did not differ according to admission time. Various theories have been postulated to explain the latter regarding the quality of care OWH, as well as other contributing factors. This has also been studied in hyperacute stroke management by telemedicine, as confirmed by a recent retrospective study that showed an increased door-to-alert time in patients consulting for AIS OWH (19). Although the “OWH effect” is an accepted phenomenon, the latter does not exclude the pertinence of telestroke management in AIS. As explored by Demaerschalk et al. in a recent publication, telestroke, defined in the cited study as a consultation involving audio and video modalities (20), remains one of the best-studied and validated models for telemedicine (21–26). An approach by telephone consultation and teleradiology by neurovascular experts has also been shown to be safe, similar to the approach adopted in HFR (27). A pooled analysis of two randomized controlled trials, including the STRokE DOC trial, comparing telemedicine and telephone only neurological assessment reported superiority of telestroke evaluation when compared with telephone alone (28, 29). Considering these results, a paradigm shift toward a system enabling distant teleneurological supervision appears appropriate in particular in limited resource settings.

Various studies exploring methods to reduce DNT exist. The systematic presence of a neurologist on site has not been incorporated in the Helsinki model published in 2012 (30) or the national initiative published by Target: Stroke, aiming to accelerate DNT in acute stroke patients (31). This study supports the appropriate use of neurological distance supervision in stroke management in certified stroke units.

Limitations of this study include the lack of documentation of other time intervals, including door-to-CT time and CT-to-needle time. Due to multiple confounding variables, the data do not allow for analysis of the role played by the neurologist in timely administration of IVT in AIS patients; further studies are necessary to address this question. A further limitation includes the difference in DNT exposed between the two centers, which may bias the comparison of patient data between GHOL and HFR. While analysis of interaction as per the ANCOVA WRS2 did not reveal a significant difference, further studies with larger population sizes would be of interest in order to minimize type II error. Lack of follow-up patient data further compromises the study quality, which is essential when evaluating the safety metrics of IVT in AIS patients. Limited data pertaining to Rankin shift, with many patients lost to follow-up, compromise evaluation of patient outcomes. Outcomes, including mortality and hemorrhagic transformation, were not systematically followed for patients who were transferred to adjacent stroke centers for potential EVT.

Patients consulting in GHOL were subject to shorter DNT irrespective of admission time (Table 3). It may be hypothesized that the 24/7 presence of a neurologist on site results in shorter DNT due to more efficient management. The latter may be justified by more uniform training of neurological and emergency stroke management teams and a possible stronger dedication to stroke care, partially illustrated by a 24/7 on-call service. While this hypothesis must be kept in mind, it is difficult to assess due to the multiple confounding variables that influence timely administration of thrombolysis. These include logistical and architectural reasons, amongst others, as previously discussed. The added value of an on-site neurologist in acute stroke management is not questioned. It is also to be noted that the quality of the diagnoses is not explored, primordial in for example stroke mimic detections or therapeutic decisions. Based on the results presented, this study suggests that telestroke may be an acceptable alternative in specific, well-defined situations. The latter involves stroke units with NIHSS-trained emergency health-care professionals. Following the change in AIS procedure in Nyon Hospital (GHOL), which shifted to telestroke management OWH in 2019, future intra-center data analysis should be undertaken to address these findings.

## Conclusion

While the time of admission modifies DNT, with a significantly longer treatment delay OWH, this study does not suggest a significant difference in DNT when comparing the presence on site of a neurologist to distance supervision with telephone communication and teleradiology. Distance supervision by a trained neurologist appears thus to be time-efficient and safe in the management of AIS. Interpretation of these conclusions requires caution in light of the retrospective nature of the analysis and the significant difference in DNT between the emergency rooms of the two stroke units considered. Nonetheless, this finding appears to support existing prospective studies in hyperacute telestroke management showing comparable DNTs between stroke and on-site neurological management.

## Data Availability Statement

The original contributions generated in the study are included in the article/supplementary material, further inquiries can be directed to the corresponding author.

## Ethics Statement

The studies involving human participants were reviewed and approved by Commission cantonale d'éthique de la recherche sur l'être humain, Av. de Chailly 23, 1012 Lausanne BASEC-ID 2019-00833. Written informed consent for participation was not required for this study in accordance with the national legislation and the institutional requirements.

## Author Contributions

FM, JN, and RS conceived the study. RS drafted the content with the contributions of all the authors. J-MA and RS conducted the statistical analysis. All authors critically revised and edited the manuscript, approving the final version.

## Conflict of Interest

The authors declare that the research was conducted in the absence of any commercial or financial relationships that could be construed as a potential conflict of interest.

## Abbreviations

BASEC: Business Administration System for Ethical Committees
DNT: door-to-needle-time
GHOL: Groupement Hospitalier de l'Ouest Lémanique
HFR: Hôpital de Fribourg
mRs: Modified Rankin Scale
NIHSS: National Institutes of Health Stroke Scale
OWH: outside of working hours
*p*: *p*-*value
r-tPA: recombinant tissue plasminogen activator
WH: working hours.
